# Stable Internal Reference Genes for Normalizing Real-Time Quantitative PCR in *Baphicacanthus cusia* under Hormonal Stimuli and UV Irradiation, and in Different Plant Organs

**DOI:** 10.3389/fpls.2017.00668

**Published:** 2017-05-03

**Authors:** Yuxiang Huang, Hexin Tan, Jian Yu, Yue Chen, Zhiying Guo, Guoquan Wang, Qinglei Zhang, Junfeng Chen, Lei Zhang, Yong Diao

**Affiliations:** ^1^School of Biomedical Sciences, Huaqiao UniversityQuanzhou, China; ^2^School of Pharmacy, Quanzhou Medical CollegeQuanzhou, China; ^3^School of Pharmacy, Second Military Medical UniversityShanghai, China; ^4^Department of Pharmacy, Changzheng Hospital, Second Military Medical UniversityShanghai, China

**Keywords:** *Baphicacanthus cusia*, hormone stimuli, plant organ, qPCR, reference gene

## Abstract

*Baphicacanthus cusia* (Nees) Bremek, the plant source for many kinds of drugs in traditional Chinese medicine, is widely distributed in South China, especially in Fujian. Recent studies about *B. cusia* mainly focus on its chemical composition and pharmacological effects, but further analysis of the plant's gene functions and expression is required to better understand the synthesis of its effective compounds. Real-time quantitative polymerase chain reaction (RT-qPCR) is a powerful method for gene expression analysis. It is necessary to select a suitable reference gene for expression normalization to ensure the accuracy of RT-qPCR results. Ten candidate reference genes were selected from the transcriptome datasets of *B. cusia* in this study, and the expression stability was assessed across 60 samples representing different tissues and organs under various conditions, including ultraviolet (UV) irradiation, hormonal stimuli (jasmonic acid methyl ester and abscisic acid), and in different plant organs. By employing different algorithms, such as geNorm, NormFinder, and BestKeeper, which are complementary approaches based on different statistical procedures, *18S* rRNA was found to be the most stable gene under UV irradiation and hormonal stimuli, whereas ubiquitin-conjugating enzyme E2 was the best suitable gene for different plant organs. This novel study aimed to screen for suitable reference genes and corresponding primer pairs specifically designed for gene expression studies in *B. cusia*, in particular for RT-qPCR analyses.

## Introduction

*Baphicacanthus cusia* (Nees) Bremek (Figure S1), widely distributed in Southern China, is the only plant belonging to the family Acanthaceae. Its roots are used as a traditional Chinese medicine, named “Nan-Ban-Lan-Gen” (National Pharmacopoeia Committee, 2015), for its antibacterial, antiviral, and immunoregulatory effects in treating colds, fever, and influenza, and especially severe acute respiratory syndrome (Sun et al., 2008). Its leaves and stems are used to extract indigo naturalis (Qingdai). Previous studies have shown that Qingdai is used to treat leukemia (Li et al., 2011; Hu et al., 2014), ulcerative colitis (Suzuki et al., 2013; Fan et al., 2014), oral cancer (Lo and Chang, 2013), and psoriasis (Lin et al., 2012). Numerous active compounds have been identified from *B. cusia* to date, such as indole alkaloids, quinazolinone alkaloids, monoterpenes, triterpenes, flavonoids, sterols, anthraquinones, benzoxazinones, and lignans. Of these ingredients, the pharmaceutical activities of indole alkaloids are most frequently reported for their leukocyte-inhibitory, anti-inflammatory, and antiviral activities (Li et al., 1993; Wu et al., 2005; Huang et al., 2014). Recently, indirubin, tryptanthrin, and isorhamnetin were successfully characterized, and their anti-leukemia effects were validated (Wu X. X. et al., 2016). These compounds are secondary metabolites synthesized during normal plant growth or in response to environmental stresses (Borowski et al., 2014). The stress response mechanism of *B. cusia* to harsh environmental conditions needs to be explored to better understand its role in producing active ingredients. At present, the genetic information of *B. cusia* for molecular biology research is limited in public databases, which makes further in-depth studies more difficult. The mRNA has recently been sequenced, and the partial contigs have already been deposited into the National Center for Biotechnology Information (NCBI) database (SRR4428209) to accelerate genetic studies in *B. cusia*.

Plants have established exceedingly complex molecular mechanisms to survive adverse environmental conditions, and the primary mechanism is induced at a transcriptional level when stresses are present (Nakashima et al., 2009; Tran et al., 2010). Moreover, studies show that most hormones can regulate plant physiological activities, and exogenous plant hormones can improve plant resistance to variable environments (Bari and Jones, 2009). For example, abscisic acid (ABA) is called “stress hormone” in plants because it plays an important role in resisting drought, salinity, and heat (Tuteja, 2007). Jasmonic acid methyl ester (MeJA) plays a key role in plant defense and growth (Zhang M. et al., 2015). Moreover, ultraviolet (UV)-B and MeJA were applied in combination for their synergistic effects on the expression levels of key genes in the biosynthetic pathway (Wang et al., 2016).

RNA sequencing (RNA-Seq), a transcriptome-based next-generation sequencing technique, has revolutionized the genome-wide gene expression analysis in various species (Wang et al., 2009; Stone and Storchova, 2015). The main outcome of RNA-Seq data is the identification of differentially expressed genes. It is also used to search for reference genes. Real-time quantitative polymerase chain reaction (RT-qPCR) is a sensitive, specific, and reproducible technique widely used to analyze the expression of genes in different organisms and tissues under different conditions (Bustin, 2002; Andersen et al., 2004; Caldana et al., 2007). Consequently, it has become the method of choice for validating candidate genes with a large sample of individuals and replicates. However, it is necessary to select suitable reference genes as internal controls under different experimental conditions for accurate RT-qPCR evaluation because of the variability in initial material, RNA integrity, RT-PCR efficiency, and RT-qPCR efficiency (Derveaux et al., 2010). Further, gene expression can be highly tissue specific and differentiated based on the physiological status of the organism or experimental treatments.

Many recent studies (Exposito-Rodriguez et al., 2008; Liu et al., 2012) have proved that it is necessary to verify the expression stability of a candidate reference gene in each species prior to its use for normalization. In this regard, several free Excel-based statistical algorithms such as geNorm (Vandesompele et al., 2002), NormFinder (Andersen et al., 2004), and BestKeeper (Pfaffl, 2001) permit the identification of the best internal controls from a set of candidate normalization genes in a given series of biological samples. They have been successfully employed to identify the most stable reference genes in animals, microorganisms, human diseases, and various plant species, such as parsley (Li M. Y. et al., 2016), *Chrysanthemum morifolium* (Qi et al., 2016), *Camellia sinensis* (Wu Z. J. et al., 2016), *Oxytropis ochrocephala* (Wang et al., 2015), and *Gentiana macrophylla* (He et al., 2016). Besides these software programs, the best suitable reference gene should be evaluated with target genes associated with experimental conditions to obtain reliable results. *B. cusia* is still a less-studied species at the molecular level. Terpenoid indole alkaloids (TIAs), derivatives of shikimate and terpenoid pathways, are important medicinal ingredients in *B. cusia*, and they are activated in response to hormonal stresses (Schluttenhofer et al., 2014; Zhang W. et al., 2015). Shikimate kinase and 1-deoxy-D-xylulose 5-phosphate reductoisomerase (DXR) are the main enzymes involved in the synthesis of TIAs (Veau et al., 2000; Kasai et al., 2005). Therefore, the genes coding for them are interesting target genes that may be able to testify the reliability of the reference genes under different experimental conditions.

The stability of 10 commonly used reference genes based on the transcriptome datasets of *B. cusia* was evaluated by RNA-Seq (unpublished data) in this study to identify potential reference genes suitable for transcript normalization in experiments under UV irradiation and hormonal stimuli (MeJA and ABA), and also in different plant organs. Moreover, the expression of two target genes, *BcSK* and *BcDXR*, was investigated to demonstrate the suitability of the selected reference genes.

## Materials and methods

### Plant material and treatments

Field-grown samples of *B. cusia* were collected from perennial dominant Shufeng Farm in Fujian, China (25°25 N 118°39C). The organ-specific series of samples (root, stem, leaf, and flower) were collected from flowering plants. Stress treatments were applied to 6-month-old plants before flowering. For UV irradiation, plants with soil were transferred into flowerpots and placed under a UV-B transilluminator (0.2 mW cm^−2^) for 3 h, and then the viable leaves were selected. The overground parts were sprayed with a solution containing either 100 μM MeJA or 100 μM ABA. Then, tissue samples, mainly comprising viable leaves, were collected at 0, 2, 4, 6, 8, 12, and 24 h after treatment. All samples were separately collected in three biological repeats. So, a total of 60 samples were analyzed, consisting of 12 organ-specific samples (root, stem, leaf, and flower) and 48 stress-treated samples (MeJA-, ABA-, and UV-treated leaves). After collection, the samples were immediately frozen in liquid N_2_ and stored at −80°C until further use.

### Total RNA and genomic DNA isolation and cDNA synthesis

The frozen samples were ground to a fine powder in liquid N_2_ using a pestle and mortar. The total RNA was extracted from all plant tissue samples using a Column Plant Total RNA Kit (TransGen Biotech, China) following the manufacturer's recommendations. The concentration of RNA samples was determined using a NanoDrop 2000 spectrophotometer (Thermo, America) at 260 nm, whereas its purity was assessed based on absorbance ratios at 260/280 nm. Samples with an optical density absorption ratio at OD260/280 between 1.9 and 2.2 and OD260/230 <2.0 were used for cDNA synthesis. The integrity of purified RNA was confirmed using agarose gel electrophoresis and ethidium bromide staining. Genomic DNA was isolated from young leaves (100 mg) using the cetyltrimethyl ammonium bromide method and checked by agarose electrophoresis. First-strand cDNAs were synthesized using the TransScript One-Step gDNA Removal and cDNA Synthesis SuperMix (TransGen Biotech, China), by adding Oligo (dT) primer, gRemover, E-mix, and R-mix to 1 μg of total RNA. RNase-free water was added to the prior mixture, and the total volume (20 μL) was incubated at 42°C for 15 min according to the manufacturer's protocol. Reverse transcriptase was inactivated by incubating the mixture at 85°C for 5 min, and the cDNA solution was stored at −20°C.

### Transcriptome data mining for candidate reference genes

Transcriptome sequencing of different *B. cusia* organs (root, stem, and leaf) was performed using the Illumina Hi-Seq 2500 platform (Illumina, America). After assembly and annotation, RNA-Seq by Expectation Maximization (Li and Dewey, 2011) was used to analyze the read counts, which were then converted into fragments per kilobase of exon per million reads mapped (FPKM) values, a commonly accepted estimate for the expression level of unigenes (Trapnell et al., 2010).

On the basis of previous studies, 10 candidate reference genes (Table 1) belonging to different functional classes were selected to avoid possible co-regulation of the genes. This group of genes comprised several classical housekeeping genes commonly used as internal control for expression studies. The sequences of candidate reference genes were obtained from the transcriptome database. The open reading frame sequences of these genes were cloned (Figure S2). The full-length cDNA sequences of the candidate reference genes are provided in Data S1. Moreover, a “tblastx” (NCBI) was run with the candidate gene sequences on non-redundant database using the default settings of the online program to identify *B. cusia* homologs. The candidate reference genes tested are listed in Table 1 with their respective reference(s) where they were first described.

**Table 1:** **Characteristics of the candidate reference genes in transcriptome datasets of *B. cusia***.

**Gene symbol**	**Gene ID**	**Gene name**	Gene length (bp)^*^	**NR description**	**NR accession number**	**ROOT_FPKM**	**STEM_FPKM**
*18S*	c65364_g1_i1	18S rRNA	5807	18S ribosomal RNA[*Neochloris vigenis*]	M74496.1	1721.05	2826.45
*CYP*	c249980_g1_i1	Cyclophilin	1212	Cyclophilin [*Momordica charantia*]	HQ171897.1	410.16	542.02
*EFa*	c104359_g2_i2	Elongation factor 1-alpha	1869	Elongation factor 1-alpha [*Chrysanthemum nankingense*]	KF305681.1	347.76	337.30
*MDH*	c127516_g1_i1	Malate dehydrogenase	1762	Malate dehydrogenase 5 [*Zea mays*]	NM_001112133.2	106.00	114.60
*TUBa*	c70526_g1_i1	Alpha-tubulin	2853	TUBA2 [*Brassica rapa*]	AB445012.1	184.56	320.28
*TUBb*	c52210_g1_i1	Beta-tubulin	3723	Tubulin [*Ornithogalum longebracteatum*]	KM370878.1	433.59	351.98
*UBC*	c187724_g1_i1	Ubiquitin-conjugating enzyme E2	1057	Ubiquitin-conjugating enzyme E2 [*Litsea cubeba*]	KF706383.1	365.88	374.90
*UBQ*	c105454_g1_i1	Ubiquitin 10	1307	Putative polyubiquitin [*Panax ginseng*]	KF680557.1	33.12	42.55
*GAPDH*	c218573_g1_i1	Glyceraldehyde-3-Phosphate dehydrogenase	2605	GAPDH [*Betula luminifera*]	KP245812.1	6.33	15.61
*ACT*	c103390_g1_i1	¡¡Actin	1827	Actin 2[*Arabidopsis thaliana*]	NM_112764.3	254.81	294.80

* *The nucleotide sequences of full-length cDNAs are deposited online (Data S1)*.

### Primer design and PCR amplification efficiency

The amplification primers for real-time PCR were designed using the Primer3 software (http://www.simgene.com/Primer3) (Rozen and Skaletsky, 2000) as a criterion to amplify products from 158 to 249 bp with a temperature of 40–60°C (primer sequences are shown in Table 2). Amplification primers were targeted to different exons to control genomic DNA contamination. The performance of the designed primers (Table 2) was tested by PCR using either *B. cusia* cDNA or genomic DNA templates (Figure S3). The presence of spurious products of amplification caused by genomic DNA was also continuously checked by verifying RT-qPCR dissociation profile. A template-free control reaction was run to ensure the absence of contamination or primer–dimer formation for each primer pair. The PCR amplification efficiency was determined for each primer combination using the slope of the standard curve obtained by plotting the fluorescence versus a given concentration of a mixture of all sample cDNAs (ranging from 1:1 to 1:10,000 dilution of the cDNA mixture sample) using the equation: *E* = 10^(−1/slope)^ – 1 (Ruijter et al., 2009), and the values were used in all subsequent analyses (Table 2).

**Table 2:** **Primer sequences of selected candidate reference genes, primers, and amplicon characteristics**.

**Name**	**Primer sequence (forward/reverse)**	**Amplicon length(bp)**	**Tm (°C)**	Primer efficiency (%)^*^	***R***^2^^*^
*GAPDH*	TTCCCAGCTCGCTCCAAAGA CTTGAGCAAGTGCGAGGCAT	199	85.7	98.1	0.9996
*18S*	GCTTCCCTCCCGACAATTTC AGTCGGGTTGTTTGGGAATG	158	84.7	89	0.9806
*EFa*	GCTTGTGACCTTTGCTCCAA CCTGGGAGGTGAAGTTAGCA	203	85.5	90.7	0.9926
*MDH*	GAGAAGTCTGTCCGTGAACT AAGAACCCAGTCACGGATGT	162	84.8	89.2	0.998
*UBQ*	AGTGCACTCATACCACCGAA CGTTCCTCCTCTAAGCCTCA	179	82.9	95.1	0.9924
*UBC*	ATGTTCCATTGGCAGGCTAC ATCGAGAGCAACACCTTGGA	230	83.3	92.3	0.992
*ACT*	CCCAAAGGCCAATCGTGAAA CGCATGGGGAAGAGCATAAC	190	83.4	88	0.9969
*TUBa*	ATCTACCCTTCTCCCCAGGT AGCCGGTTGAGATTGGTGTA	179	86.8	87.5	0.9905
*TUBb*	GAAGATCAAAGATGAGAGAA AACACAGCCCTCGGGACATA	205	85.5	90.9	0.9946
*CYP*	CGCGTGATCCCGAATTTCAT GTCTAAGCCCTCGACAACCT	249	89.1	91.9	0.9826

* *Mean of 3 technical replicates*.

### Real-time quantitative polymerase chain reaction

Real-time amplification reactions were performed using SYBR Green detection chemistry and run in 96-well plates with the Thermal Cycler Dice TP800 (TaKaRa, Japan). Reactions were performed in a total volume of 20 μL containing 2.0 μL of template, 0.5 μL of each amplification primer, 10.0 μL of 2 × Top Green qPCR SupperMix (TransGen Biotech, China), and 7.0 μL of ddH_2_O. All reaction components without template were used as a negative control. The amplification program was set as follows: initial denaturation step of 95°C for 30 s to activate the DNA polymerase, followed by 40 cycles of denaturation at 95°C for 5 s and annealing at 60°C for 30 s. The amplification process was followed by a melting curve analysis, ranging from 60 to 95°C. Amplicon-based fluorescence thresholds were used to obtain the *Ct*-values. All RT-qPCR reactions were carried out in triplicate, both technically and biologically. The final quantification cycle (*Cq*) values were the mean of nine values (biological triplicate, each in technical triplicate). Melting curve analysis of the amplification products and gel electrophoresis analysis confirmed that the primers amplified only a single product (data not shown).

The expression of target genes *BcSK* and *BcDXR*, which were important in the synthesis of compounds in *B. cusia*, was analyzed using the selected reference genes to validate the reference gene selection. The changes in relative expression levels were calculated using the 2^ΔΔCt^ method (Pfaffl, 2001). Furthermore, the expression levels of both were compared with the FPKM values in RNA-Seq data in some samples (Table S1).

### Statistical analysis of gene expression stability

The suitability of candidate control genes across all the experimental sets was evaluated using three statistical algorithms, geNorm (version 3.5) (Vandesompele et al., 2002), NormFinder (Andersen et al., 2004), and BestKeeper (Pfaffl, 2001) programs, according to their respective protocols. For geNorm and NormFinder, the raw *Ct*-values of each gene were converted into the relative quantities using the formula 2^−ΔCt^ (ΔCt = each corresponding *Ct*-value – lowest *Ct*-value). For BestKeeper, the *Ct*-value in the program was used directly. Genes with standard deviation (SD) >1 were considered to be unacceptable as reference genes. The pairwise variation (*V*_*n*_/*V*_*n*+1_) was calculated additionally using the geNorm software to determine the optimal number of reference genes needed to normalize; additional control gene was not required for normalization when *V* was <0.15 (Vandesompele et al., 2002).

The 60 samples were divided into 6 experimental sets and analyzed individually to achieve a more accurate expression analysis. Set 1 comprised 21 samples from the MeJA-induced *B. cusia* leaves, set 2 comprised 21 samples from the ABA-induced *B. cusia* leaves, set 3 comprised 6 samples from the UV-irradiated *B. cusia* leaves, set 4 comprised12 samples from different organs (root, stem, leaf, and flower) of *B. cusia*, and set 5 which was defined as the stress group comprised samples from sets 1, 2, and 3. Also, the overall stability among the five sets and every variety was analyzed.

## Results

### Selecting reference genes based on transcriptome datasets

A total of 10 genes were selected as candidate genes with their FPKM values, as shown in Table 1. They were glyceraldehyde-3-phosphate dehydrogenase (*GAPDH*), malate dehydrogenase (*MDH*), ubiquitin 10 (*UBQ*), ubiquitin-conjugating enzyme E2 (*UBC*), actin (*ACT*), 18S rRNA (*18S*), elongation factor 1-alpha (*EFa*), cyclophilin (*CYP*), alpha-tubulin (*TUBa*), and beta-tubulin (*TUBb*) housekeeping genes, and these genes have all been reported as stable genes in other species. The sequences of the 10 reference genes were obtained from the transcriptome database (Data S1). Gene characteristics are shown in Table 1. The full-length unigene sequences from transcriptome database, which were used to design the specific primers for RT-qPCR, could be obtained in NCBI (accession number SRR4428209).

### Performance of amplification primers

The specificity and efficiency of each primer pair were assessed by amplification and dissociation curve analysis. First, the performance of the amplification primers was tested by real-time PCR using cDNA and gDNA as templates, respectively. A single band with the expected size (Table 2) was obtained in each case without signs of primer–dimer formation (Figure S3), and four primer pairs yielded amplicons longer than those obtained with a cDNA template (Figure S3), whereas primers for other genes were unable to amplify genomic sequences. Second, the melting curve analysis of the amplification products was performed by qPCR after 40 cycles of amplification. The presence of a single peak indicated that the expected amplicons were amplified (Figure S4). The amplicons were also examined by 1% agarose gel electrophoresis using ethidium bromide staining, and a single band of the expected size for each primer pair was observed. The correlation coefficients (*R*^2^) ranged between 0.9826 and 0.9996, and PCR amplification efficiencies between 87.5 and 98.1% (Table 2); both were results of three technical replicas. No signals were detected in no-template controls.

### Expression stability of reference genes

**Figure 1:**
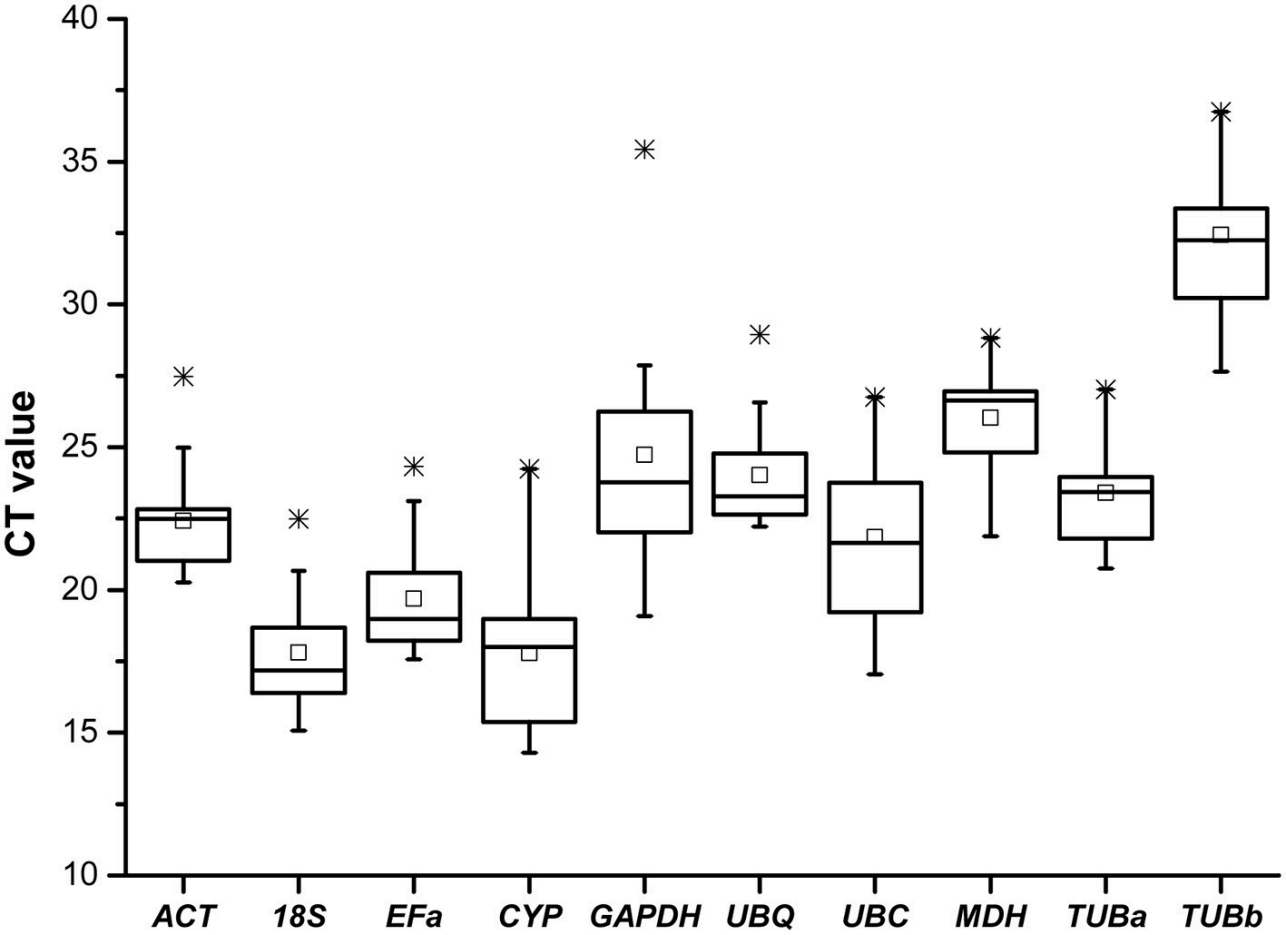
**Cycle threshold (*Ct*) values of 10 candidate reference genes across all samples**. The final *Ct*-value of each sample was the mean of three biological and technical replicates. Box graph indicates the interquartile range. A line across the box is shown as the median. Lower and upper dashes represent the minimum and maximum values, respectively; middle panes show the mean values. ^*^Represents the extremum value.

Real-time RT-PCR was conducted on the 60 cDNA samples with 10 primer pairs. The 10 candidate control genes displayed a relatively wide range of expression level with mean *Ct*-values between 14.3 (*CYP*) and 36.5 (*TUBb*) (Figure 1), suggesting that these reference genes were expressed at different levels in *B. cusia*. The genes with higher SD of *Ct*-values indicated more variable expression compared with those with lower SD, as shown in **Table 4**.

### geNorm analysis

**Figure 2:**
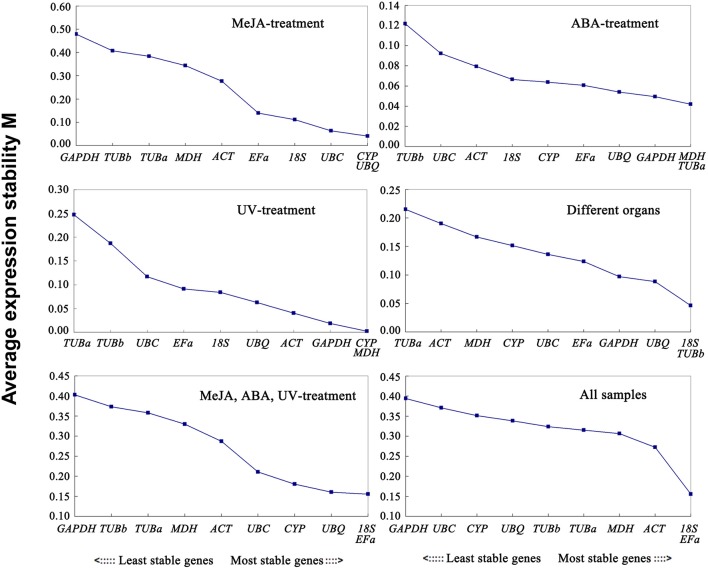
**Expression stability values of 10 candidate reference genes calculated by geNorm**. Lower *M*-values indicate more stable expression. Ranking of the gene expression stability was performed in all subset samples. The least stable genes were on the left and the most stable genes on the right.

The geNorm program classifies the stability of gene expression by calculating the average expression stability (*M*). Stably expressed genes have values below 1.5, and an *M*-value more than 1.5 indicates lower expression stability (Vandesompele et al., 2002). The ranking order according to the *M* value is depicted in Figure 2. As determined by geNorm, *18S* and *EFa* were the most stable reference genes in total samples. In contrast, *GAPDH* and *UBC* were the least stable reference genes. Under each subset, the two best reference genes in MeJA stress were *CYP* and *UBQ* with the lowest *M*-value. The most preferred genes for normalization in the ABA stress were *MDH* and *TUBa*. The two most stable reference genes in UV stress were *CYP* and *MDH*. As for the various organs, *18S* and *TUBb* were the most stable reference genes. Furthermore, the best stable control genes in the stress group (set 5) were *18S* and *EFa*.

**Figure 3:**
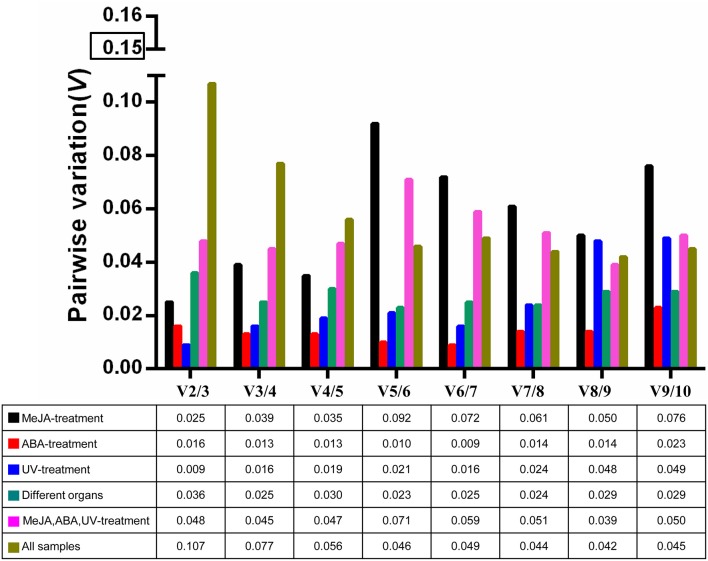
**Pairwise variation (*V*) of 10 candidate reference genes calculated by geNorm to determine the optimal number of reference genes for accurate normalization**. The threshold is 0.15.

The optimal number of reference genes required for accurate normalization was determined by the pairwise variation (*V*). The *V*_*n*_/*V*_*n*+1_ values were below 0.15 under all experimental conditions (Figure 3), indicating that one stable reference gene was enough to obtain accurate results.

### NormFinder analysis

The NormFinder program is another visual basic application tool for Microsoft Excel used to determine expression stabilities of reference genes. It ranks genes based on the stability value for each reference gene. More stable gene expression has the lower stability value. Moreover, this algorithm suggests the best pair of genes among the candidate reference genes analyzed using the intra- and intergroup variance. The stability values of reference genes were calculated by NormFinder, as shown in Table 3. In the subset of MeJA stress, *18S* and *EFa* were the most stable. In ABA stress, *MDH* and *GAPDH* were the most stable. In UV stress, the top two stably expressed genes were *CYP* and *MDH*. In different normal organs (non-stress group), *UBC* and *EFa* were the top two stably expressed genes. In set 5, *EFa* and *UBQ* were the most highly ranked. When evaluating the total experimental samples, *UBQ* and *18S* were the top ranked genes (Table 3). In contrast, *GAPDH* was the least stable reference gene under MeJA stress, *TUBb* was the least stable under ABA stress, and *TUBa* was the least stable under UV irradiation and normal organs. *TUBa* was also the least stably expressed gene in set 5 and the second least stably expressed in total samples. The rank in NormFinder was slightly different from that in geNorm. Genes considered as the most stable by geNorm (*18S* and *EFa*) ranked second and third by NormFinder, respectively.

**Table 3:** **Candidate genes ranked according to their expression stability as determined by NormFinder**.

**Rank**	**MeJA-treatment**		**ABA-treatment**		**UV-treatment**		**Different organs**		**MeJA, ABA,UV-treatment**		**All samples**	
	**Gene**	**Stability**	**Gene**	**Stability**	**Gene**	**Stability**	**Gene**	**Stability**	**Gene**	**Stability**	***Gene***	**Stability**
1	*18S*	0.039	*MDH*	0.015	*CYP*	0.001	*UBC*	0.034	*EFa*	0.080	*UBQ*	0.090
2	*EFa*	0.039	*GAPDH*	0.017	*MDH*	0.001	*EFa*	0.067	*UBQ*	0.094	*18S*	0.095
3	*UBC*	0.137	*TUBa*	0.022	*GAPDH*	0.009	*18S*	0.079	*18S*	0.101	*EFa*	0.097
4	*CYP*	0.186	*CYP*	0.029	*ACT*	0.016	*CYP*	0.088	*GAPDH*	0.111	*GAPDH*	0.113
5	*UBQ*	0.211	*UBQ*	0.038	*UBQ*	0.068	*MDH*	0.097	*ACT*	0.119	*ACT*	0.136
6	*ACT*	0.261	*EFa*	0.039	*18S*	0.114	*UBQ*	0.098	*CYP*	0.154	*CYP*	0.148
7	*MDH*	0.300	*18S*	0.050	*EFa*	0.117	*TUBb*	0.098	*TUBb*	0.182	*TUBb*	0.152
8	*TUBa*	0.337	*ACT*	0.066	*UBC*	0.199	*GAPDH*	0.136	*UBC*	0.182	*MDH*	0.169
9	*TUBb*	0.353	*UBC*	0.101	*TUBb*	0.254	*ACT*	0.164	*MDH*	0.191	*TUBa*	0.189
10	*GAPDH*	0.524	*TUBb*	0.162	*TUBa*	0.336	*TUBa*	0.197	*TUBa*	0.202	*UBC*	0.205

### BestKeeper analysis

The BestKeeper algorithm evaluates the stabilities of candidate reference genes based on the CV ± SD values. In the MeJA stress set, *18S* (2.65 ± 0.36) and *UBQ* (1.85 ± 0.46) with lowest CV ± SD values were somewhat similar to the results of geNorm and NormFinder. In the ABA stress set, *MDH* (0.78 ± 0.21) and *UBQ* (0.98 ± 0.22) were considered as the most stable genes. In the UV stress treatment, *18S* (0.11 ± 0.02) and *EFa* (0.22 ± 0.04) were identified as the best reference genes for normalization. As for the subsequent three subsets, *18S* was the best stable gene, which was consistent with the result of geNorm (Table 4).

**Table 4:** **Expression stability of 10 candidate reference genes calculated by BestKeeper**.

**Rank**	**MeJA-treatment**			**ABA-treatment**			**UV-treatment**			**Different organs**			**MeJA, ABA,UV-treatment**			**All samples**		
	**Gene**	**SD**	**CV**	**Gene**	**SD**	**CV**	**Gene**	**SD**	**CV**	**Gene**	**SD**	**CV**	**Gene**	**SD**	**CV**	**Gene**	**SD**	**CV**
1	*18S*	0.36	2.65	*MDH*	0.21	0.78	*18S*	0.02	0.11	*18S*	0.89	6.04	*18S*	0.30	2.22	*18S*	0.53	3.93
2	*UBQ*	0.46	1.85	*UBQ*	0.22	0.98	*EFa*	0.04	0.22	*TUBb*	1.16	3.52	*ACT*	0.90	4.07	*ACT*	1.07	4.75
3	*CYP*	0.48	2.59	*TUBa*	0.24	1.00	*ACT*	0.37	1.77	*CYP*	1.74	8.33	*TUBa*	0.96	4.11	*TUBa*	1.16	4.97
4	*UBC*	0.64	2.59	*18S*	0.24	1.83	*UBQ*	0.46	1.99	*MDH*	1.76	6.93	*UBQ*	1.01	4.27	*UBQ*	1.36	5.65
5	*MDH*	0.74	2.80	*EFa*	0.39	2.07	*UBC*	0.66	3.71	*EFa*	1.87	8.78	*MDH*	1.13	4.32	*MDH*	1.40	5.37
6	*ACT*	0.95	4.26	*CYP*	0.47	3.11	*MDH*	0.71	3.14	*TUBa*	1.87	8.05	*EFa*	1.19	6.16	*EFa*	1.44	7.33
7	*EFa*	1.22	5.97	*ACT*	0.58	2.59	*CYP*	0.98	5.35	*UBQ*	1.96	7.58	*CYP*	1.53	8.97	*TUBb*	1.84	5.68
8	*TUBa*	1.34	5.72	*UBC*	0.79	3.71	*TUBa*	2.20	9.57	*UBC*	1.97	9.95	*GAPDH*	1.91	8.13	*CYP*	1.87	10.54
9	*GAPDH*	1.35	5.39	*GAPDH*	1.38	6.21	*TUBb*	2.54	7.92	*ACT*	1.98	8.40	*TUBb*	2.01	6.23	*UBC*	2.32	10.61
10	*TUBb*	2.02	5.99	*TUBb*	1.47	4.77	*GAPDH*	3.59	15.83	*GAPDH*	4.90	16.59	*UBC*	2.10	9.41	*GAPDH*	2.92	11.81

### Assessment of normalization in sample subsets

**Figure 4:**
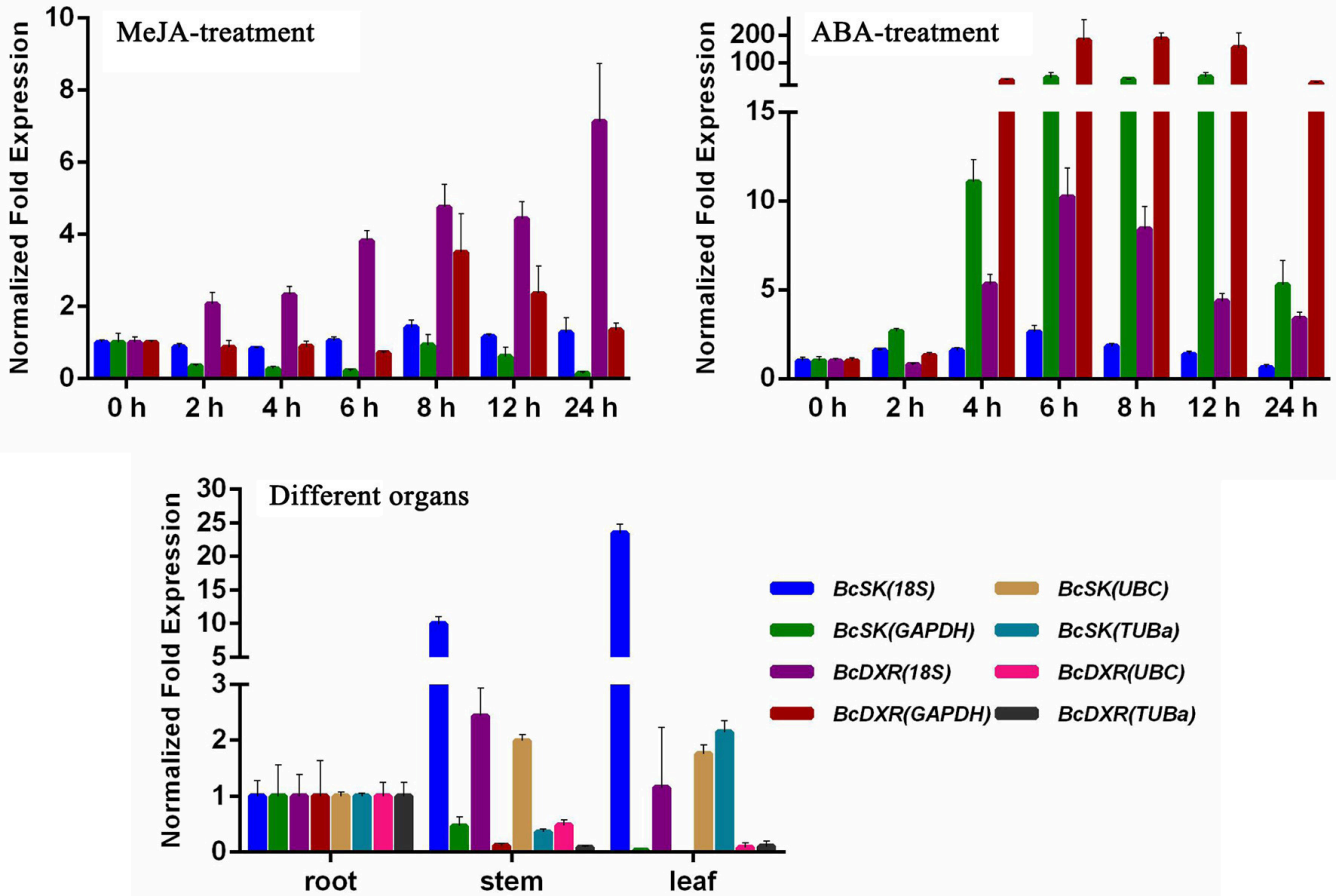
**Relative expression patterns of *BcSK* and *BcDXR* under hormonal stimuli and organs**. *18S, UBC, GAPDH*, and *TUBa* were used as reference genes for expression normalization.

The comprehensive rankings of the candidate reference genes were determined using the RefFinder program online (http://fulxie.0fees.us/?type=reference). Data generated by the three programs across different experimental sets were further compared, as shown in Table 5. The top- and low-ranked candidate reference genes were selected for normalizing two target genes *BcSK* and *BcDXR* under different experimental conditions. The result (Figure 4) showed that the expression level of *BcSK* and *BcDXR* in *B. cusia* under MeJA treatment increased with the increase in induction time when *18S* was used as a control but attained a different expression pattern with the least stable gene *GAPDH*. In ABA stress, the expression level of *BcSK* and *BcDXR* reached the highest at 6 h when using the most stable reference genes (*18S*) as the internal control, while the expression level was overestimated when the least stable genes were used (*GAPDH*). Similarly, the relative expression level of *BcSK* increased when normalized using the most stable genes (*18S*) in normal organs, while the expression level was overestimated when normalized using the least stable combination (*GAPDH*). The relative expression patterns of *BcDXR* were opposite when the foregoing two genes were used as controls. Meanwhile, *UBC* and *TUBa*, which showed the most and least stable genes, respectively, in different organs subset were used as the reference genes, and the expression levels of *BcDXR* and *BcSK* were generally identified with the expression profile in RNA-Seq (Figure 4). The result showed that *UBC* was more suitable for organs in *B. cusia*.

**Table 5:** **Expression stability ranking of the 10 candidate reference genes**.

**Method/Rank**	**1**	**2**	**3**	**4**	**5**	**6**	**7**	**8**	**9**	**10**
**MeJA-TREATMENT**										
Recommended comprehensive ranking	*18S*	*EFa*	*UBQ*	*CYP*	*UBC*	*ACT*	*MDH*	*TUBa*	*TUBb*	*GAPDH*
GeNorm	*CYP/UBQ*		*UBC*	*18S*	*EFa*	*ACT*	*MDH*	*TUBa*	*TUBb*	*GAPDH*
NormFinder	*18S*	*EFa*	*UBC*	*CYP*	*UBQ*	*ACT*	*MDH*	*TUBa*	*TUBb*	*GAPDH*
Bestkeeper	*18S*	*UBQ*	*CYP*	*UBC*	*MDH*	*ACT*	*EFa*	*TUBa*	*GAPDH*	*TUBb*
**ABA-TREATMENT**										
Recommended comprehensive ranking	*MDH*	*UBQ*	*TUBa*	*GAPDH*	*EFa*	*CYP*	*18S*	*ACT*	*UBC*	*TUBb*
GeNorm	*MDH/TUBa*		*GAPDH*	*UBQ*	*EFa*	*CYP*	*18S*	*ACT*	*UBC*	*TUBb*
NormFinder	*MDH*	*GAPDH*	*TUBa*	*CYP*	*UBQ*	*EFa*	*18S*	*ACT*	*UBC*	*TUBb*
Bestkeeper	*MDH*	*UBQ*	*TUBa*	*18S*	*EFa*	*CYP*	*ACT*	*UBC*	*GAPDH*	*TUBb*
**UV-TREATMENT**										
Recommended comprehensive ranking	*CYP*	*MDH*	*GAPDH*	*ACT*	*UBQ*	*18S*	*EFa*	*UBC*	*TUBb*	*TUBa*
GeNorm	*CYP/MDH*		*GAPDH*	*ACT*	*UBQ*	*18S*	*EFa*	*UBC*	*TUBb*	*TUBa*
NormFinder	*CYP*	*MDH*	*GAPDH*	*ACT*	*UBQ*	*18S*	*EFa*	*UBC*	*TUBb*	*TUBa*
Bestkeeper	*18S*	*EFa*	*ACT*	*UBQ*	*UBC*	*MDH*	*CYP*	*TUBa*	*TUBb*	*GAPDH*
**DIFFERENT ORGANS**										
Recommended comprehensive ranking	*UBC*	*18S*	*CYP*	*EFa*	*TUBb*	*UBQ*	*MDH*	*GAPDH*	*ACT*	*TUBa*
GeNorm	*18S/TUBb*		*UBQ*	*GAPDH*	*EFa*	*UBC*	*CYP*	*MDH*	*ACT*	*TUBa*
NormFinder	*UBC*	*EFa*	*18S*	*CYP*	*MDH*	*UBQ*	*TUBb*	*GAPDH*	*ACT*	*TUBa*
Bestkeeper	*18S*	*TUBb*	*CYP*	*MDH*	*EFa*	*TUBa*	*UBQ*	*UBC*	*ACT*	*GAPDH*
**MeJA, ABA,UV-TREATMENT**										
Recommended comprehensive ranking	*18S*	*UBQ*	*ACT*	*EFa*	*TUBa*	*MDH*	*CYP*	*GAPDH*	*TUBb*	*UBC*
GeNorm	*18S/EFa*		*UBQ*	*CYP*	*UBC*	*ACT*	*MDH*	*TUBa*	*TUBb*	*GAPDH*
NormFinder	*EFa*	*UBQ*	*18S*	*GAPDH*	*ACT*	*CYP*	*TUBb*	*UBC*	*MDH*	*TUBa*
Bestkeeper	*18S*	*ACT*	*TUBa*	*UBQ*	*MDH*	*EFa*	*CYP*	*GAPDH*	*TUBb*	*UBC*
**ALL SAMPLES**										
Recommended comprehensive ranking	*18S*	*UBQ*	*EFa*	*ACT*	*TUBa*	*MDH*	*TUBb*	*CYP*	*UBC*	*GAPDH*
GeNorm	*18S/EFa*		*ACT*	*MDH*	*TUBa*	*TUBb*	*UBQ*	*CYP*	*UBC*	*GAPDH*
NormFinder	*UBQ*	*18S*	*EFa*	*GAPDH*	*ACT*	*CYP*	*TUBb*	*MDH*	*TUBa*	*UBC*
Bestkeeper	*18S*	*ACT*	*TUBa*	*UBQ*	*MDH*	*EFa*	*TUBb*	*CYP*	*UBC*	*GAPDH*

## Discussion

RT-qPCR has become a broadly accepted method of choice for accurate expression profiling of target genes in the gene expression analysis (Nolan et al., 2006; VanGuilder et al., 2008). The accurate expression level of a selected gene requires appropriate internal controls, which are commonly called housekeeping or reference genes (Derveaux et al., 2010). The ideal control genes should be stably expressed under each experimental condition, independent of organs, tissues, developmental stages, and different treatments (Bustin et al., 2009). However, no universally suitable control genes are available, since numerous studies have reported that the expression of reference genes can also vary considerably with experimental conditions and different species (Reid et al., 2006; Wan et al., 2011; Galli et al., 2013; Li J. et al., 2016). Therefore, a set of potential housekeeping genes must be previously validated in each particular experimental subset. To date, few studies have compared and selected housekeeping genes in *B. cusia*. This has hindered the characterization of genes involved in different organisms and stress responses in *B. cusia*. This novel study performed the first large-scale transcriptome data analysis for *B. cusia* (unpublished), consisting of 293,666 unigenes, which were used for reference gene selection. The RNA-Seq data were useful sources for screening candidate housekeeping genes and represented an important strategy for large-scale reference gene selection for non-model plants (Zhuang et al., 2015).

Traditional genes, which are involved in cytoskeleton structure (*ACT, TUBa*, and *TUBb*), protein synthesis (*EF*α and *18S*), biological metabolic processes (*GAPDH* and *UBQ*), and multifunctional proteins (*CYP* and *MDH*), are usually used as reference genes. In this study, the foregoing 10 common internal control genes of *B. cusia* were cloned (Figure S2) for expression normalization in 60 different samples, including UV irradiation, hormonal stimuli (MeJA and ABA), and different organs. This novel study reported a systematic analysis of reference genes that could be used in different treatment samples of *B. cusia*. The result demonstrated that each species might have its own suitable reference genes, which should be determined for each subset of experimental conditions.

Three different, yet complementary, statistical programs were used to identify the most suitable internal controls for the normalization of gene expression studies in *B. cusia* to minimize bias entrapped by the statistical approach. The results obtained from geNorm, NormFinder, and BestKeeper were not completely identical due to the different calculation programs (Jacob et al., 2013), especially under specific individual conditions. Similar findings were reported in other studies on rice (Kim et al., 2003), coffee (Cruz et al., 2009), tobacco (Delaney, 2010), and parsley (Li M. Y. et al., 2016). Furthermore, the RefFinder program (Xie et al., 2012) was used to generate a comprehensive ranking of candidate reference genes. As a result, *18S* rRNA was the most stable gene in all subsets except ABA stimuli and UV irradiation, while *GAPDH, TUBa*, and *TUBb* yielded poor values in this study.

Reference genes varied in different tissues, organs, and experimental conditions in most previous studies. In rice, *UBQ5* and *EFa* were the most stable genes in all the tissue samples, while *18S* and *25S* rRNAs were the most stable genes under various treatment conditions (Jain et al., 2006). However, another study showed that *18S* and *25S* rRNAs had the least stable expression at different developmental stages and in different varieties of rice (Li et al., 2010). Moreover, the selections of reference genes were not consistent in various species. *ACT* and *TUBb* in carrot (Tian et al., 2015) were the most suitable reference genes under “abiotic stress” and “hormonal stimuli,” but they were not suitable choices in the present study. Moreover, *GAPDH* showed a good performance in “hormonal stimuli” in parsley (Li M. Y. et al., 2016) but was the least stable in carrot. Also, *GAPDH* was the least stable gene in *Eucalyptus* spp. (Boava et al., 2010) and *Petunia hybrida* (Mallona et al., 2010). The results demonstrated that each species might choose its own stably expressed genes for each subset of experimental conditions. *18S* rRNA is frequently used as the control gene for normalizing because it is independent of developmental stages and external stimuli (Gantasala et al., 2013). Moreover, the 26 reports (real-time RT-PCR carried out on barley; published in the period of January 1996 to March 2008) examined indicated that *18S* rRNA was the most frequently used gene (8 times) among the 16 different reference genes used (Kozera and Rapacz, 2013).

The expression of *BcSK* and *BcDXR* genes was quantified in this study. *BcSK* and *BcDXR* were found to play important roles in the indole and carotenoid biosynthesis pathways, respectively. GeNorm is used in most studies because of its capacity to determine the number of genes necessary for normalization (Kozera and Rapacz, 2013). The pairwise variation (*V*) was analyzed in this study to determine the optimal number of genes required for normalization; the *V*_*n*_/*V*_*n*+1_ values were below 0.15 under all subset conditions (Figure 3), indicating that one stable reference gene was enough to obtain accurate results. Therefore, *18S* rRNA and *GAPDH* were used to testify the expression levels of *BcSK* and *BcDXR* under stress conditions and different plant organs. The expression levels of *BcSK* and *BcDXR* increased with induction time in the MeJA treatment subset, verifying that TIAs are also induced by MeJA in *B. cusia*. Moreover, the more the expression of *BcSK*, which participates in the shikimate pathway, the higher the production of indigo (Figure S5). However, it decreases later because of branching to other aromatic compounds. Both the target genes had a peak expression at 6 h in the ABA stimuli subset as a result of the plant resistance to stress, conforming to the phenomenon that ABA not only promoted withering of an organism but could also improve disease resistance in plants. The expression pattern of both genes was generally identified with the expression profile in RNA-Seq in different plant organs.

In general, *18S* rRNA is the best gene among all samples, whereas *UBC* is the most suitable gene for plant organs, thereby facilitating future studies on gene expression in *B. cusia*. However, normalization with multiple reference genes has become the commonly used method to avoid erroneous data that may be triggered by using a single reference gene. Therefore, based on transcriptome datasets, more novel and stable reference genes can be identified from other *B. cusia* samples in further studies.

## Author contributions

YH, HT, LZ, and YD conceived and designed the study. YH, JY, GW, and YC collected the tissue material and performed the experiments. YH, JC, ZG, and QZ performed data analysis. YH wrote the manuscript. All authors read and approved the final manuscript.

### Conflict of interest statement

The authors declare that the research was conducted in the absence of any commercial or financial relationships that could be construed as a potential conflict of interest.

## Supplementary material

Figure S1 **Photograph of *B. cusia***.

Figure S2 **The amplification of ORF regions of candidate genes**.

Figure S3 **Performance of the amplification primers**. Amplicons obtained by real-time PCR using cDNA (odd numbers) or gDNA (even numbers) as template, separated by agarose gel electrophoresis.

Figure S4 **Melting curves generated for 10 candidate reference genes by qPCR in *B. cusia***.

Figure S5 **Indigo content in leaves of *B. cusia* after MeJA induction**.

Table S1 **FPKM values of the target genes in transcriptome datasets of *B. cusia***.

Data S1 **The full-length cDNA sequences of candidate genes. The ORF regions were highlighted in blue color**.
