# Supplementary material for: Stable Internal Reference Genes for Normalizing Real-Time Quantitative PCR in Baphicacanthus cusia under Hormonal Stimuli and UV Irradiation, and in Different Plant Organs
Source: Front Plant Sci. 2017 May 3;8:668. doi: 10.3389/fpls.2017.00668 (PMC5413499; doi:10.3389/fpls.2017.00668)
Supplement: Figure S1 — Photograph of B. cusia. [file DataSheet1.DOCX]

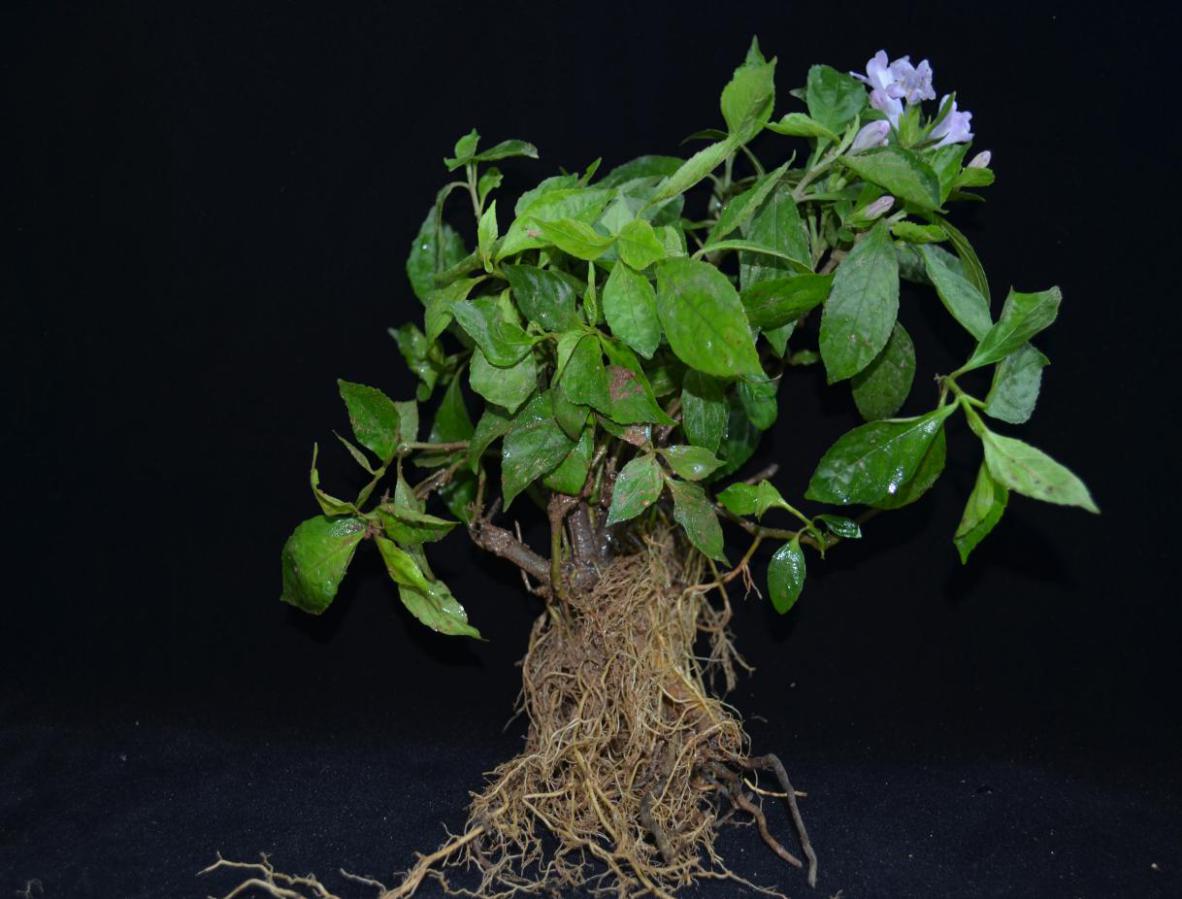


Figure S1. Photograph of *B.cusia*.


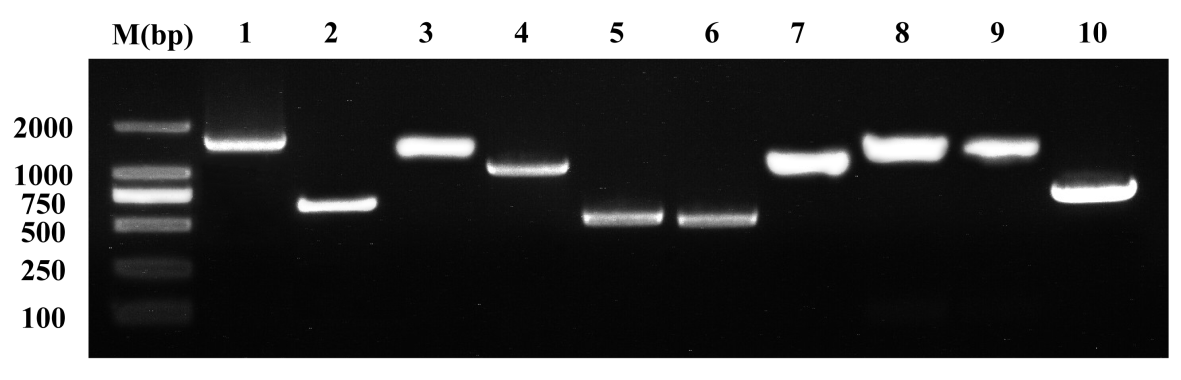


Figure S2. The amplification of ORF regions of candidate genes. Bands were targeted to *GAPDH* (1), *18S* (2), *EFa* (3), *MDH* (4), *UBQ* (5), *UBC* (6), *ACT* (7), *TUBa* (8), *TUBb* (9), *CYP* (10).


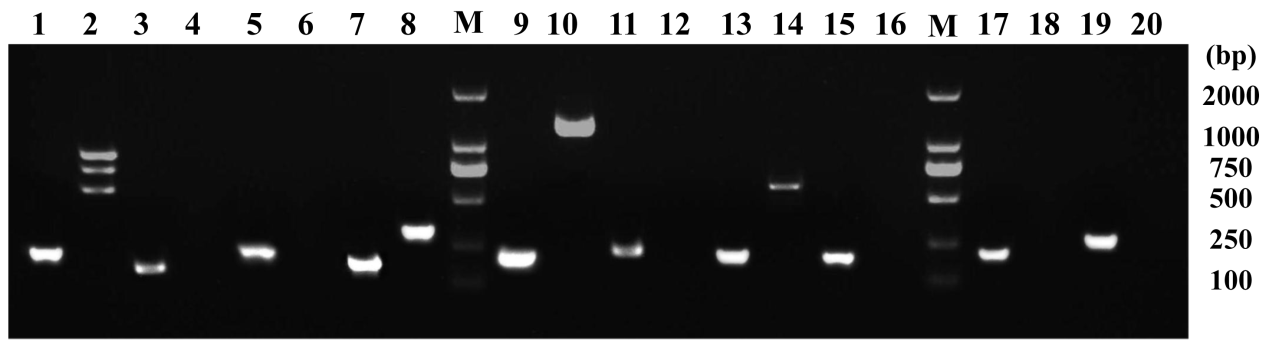


Figure S3. Performance of the amplification primers. Amplicons obtained by real-time PCR using cDNA (odd numbers) or gDNA (even numbers) as template, separated by agarose gel electrophoresis. Amplification primers were targeted to *GAPDH* (1–2), *18S* (3–4), *EFa* (5–6), *MDH* (7–8), *UBQ* (9–10), *UBC* (11–12), *ACT* (13–14), *TUBa* (15–16), *TUBb* (17–18), *CYP* (19–20).


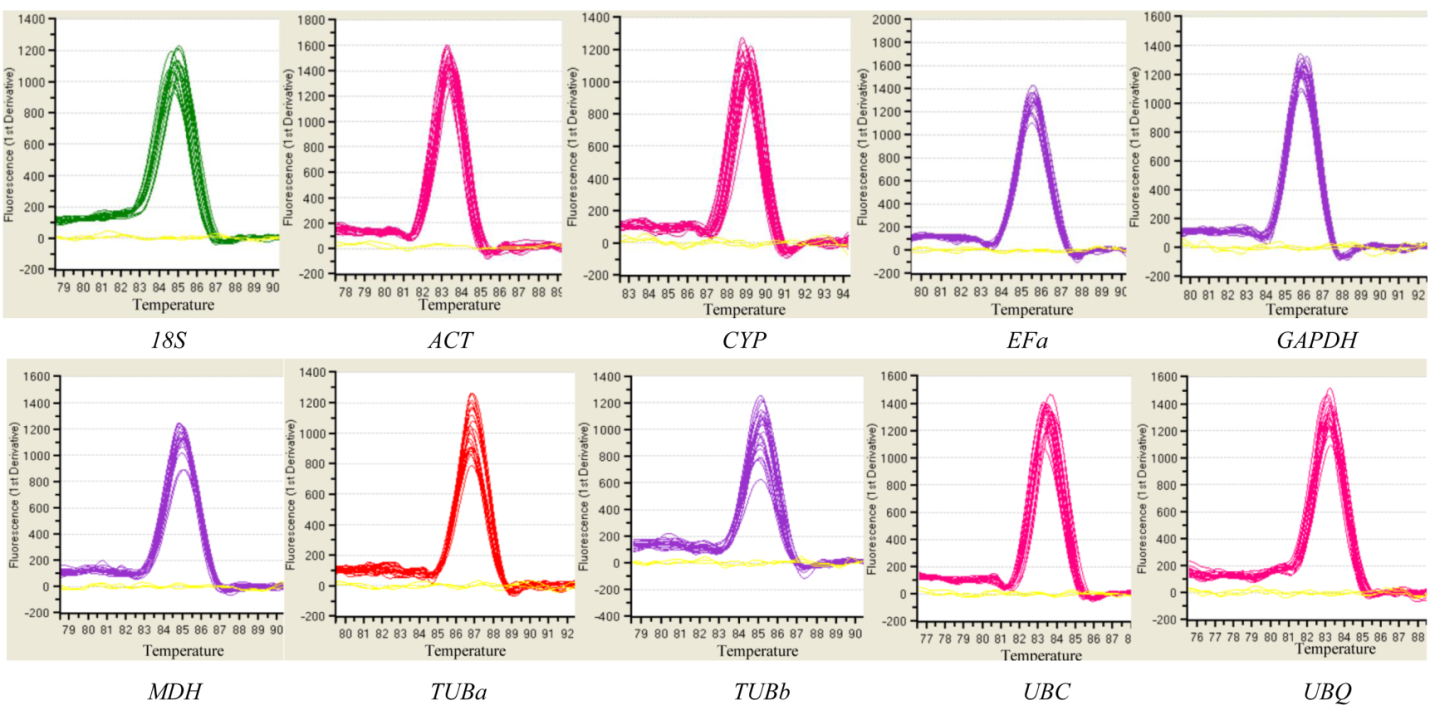


Figure S4. Melting curves generated for 10 candidate reference genes by qPCR in *B. cusia*.





Figure S5. The indigo content in leaves of *B. cusia* after MeJA induction.
