# Supplementary material for: Stable Internal Reference Genes for Normalizing Real-Time Quantitative PCR in Baphicacanthus cusia under Hormonal Stimuli and UV Irradiation, and in Different Plant Organs
Source: Front Plant Sci. 2017 May 3;8:668. doi: 10.3389/fpls.2017.00668 (PMC5413499; doi:10.3389/fpls.2017.00668)
Supplement: Data S1 — The full-length cDNA sequences of candidate genes. The ORF regions were highlighted in blue color. [file DataSheet2.DOC]

>*18S* c65364_g1_i1

ggctgaatctcagcggatcgtggcagcaaggccactctgccgcttacaataccccgtcgcgtatttaagtcgtctgcaaaggattctacccgccgctcgatgggaattgtaattcaaggcggcccacgcggctcgtccgccgcgagggcttggcccacgacacgtgcccttgggggccaaaaggcccctactgcgggtcggcaagcggacggcgggcatacgcatcgcttctagcccggattctgacttagaggcgttcagtcataatccagcgcacggtagcttcgcgccactggcttttcaaccaagcgcgatgaccaattgtgcgaatcaacggttcctctcgtactaggttgaattactattgcgacactgtcatcagtagggtaaaactaacctgtctcacgacggtctaaacccagctcacgttccctattggtgggtgaacaatccaacacttggtgaattctgcttcacaatgataggaagagccgacatcgaaggatcaaaaagcaacgtcgctatgaacgcttggctgccacaagccagttatccctgtggtaacttttctgacacctctagcttcaaattccgaaggtctaaaggatcgttaggccacgctttcacggttcgtattcgtactggaaatcagaatcaaacgagcttttacccttctgttccacacgagatttctgttctcgttgagctcatcttaggacacctgcgttatcttttaacagatgtgccgccccagccaaactccccacctgacaatgtcttccgcccggatcggcccgccgaggcgagccttgggtccaaaaagaggggcgatgccccgcctccgattcacggaataagtaaaataacgttaaaagtagtggtatttcactttcgcctttcggctcccacttatcctacacctctcaagtcatttcacaaagtcggactagagtcaagctcaacagggtcttctttccccgctgattccgccaagcccgttcccttggctgtggtttcgctggatagtagacagggacagtgggaatctcgttaatccattcatgcgcgtcactaattagatgacgaggcatttggctaccttaagagagtcatagttactcccgccgtttacccgcgcttggttgaatttcttcactttgacattcagagcactgggcagaaatcacattgcgtgagcatccgcagggaccatcgcaatgctttgttttaattaaacagtcggattccccttgtccgtaccagttctgagtcgactgttcgacgcccggggaaggcccccgcgaaggagccgttcccagtccgtcccccggccggcacgcgacgacccgctctcgccacggtagcagctcgagcagttcgccgacagccgacgggttcgggactgggacccccgtgcccagccctcagagccaatccttttcccgaggttacggatccattttgccgacttcccttgcctacattgttccatcgaccagaggctgttcaccttggagacctgatgcggttatgagtacgaccgggcgcggacggcactcggtcctccggattttcaagggccgccgggggcgcaccggacaccacgcgacgtgcggtgctcttccagccgctggaccctacctccggctgagccgtttccagggtgggcaggctgttaaacagaaaagataactcttcccgaggcccccgccggcgtctccggactccctaacgttgccgtcagccgccgcgtcccggttcaggaattttaacccgattccctttcggcgcacgcgcggaacgcgctgtctgtcgggtttcccccgacccttaggatcgactaacccatgtgcaagtgccgttcacatggaacctttcccctcttcggccttcaaagttctcatttgaatatttgctactaccaccaagatccgcaccgacggccgctccgcccgggcttgcgcccaaggttttgcagcgaccgccgcgccctcctactcatcggggcctggctcttgccccgacggccgggtataggtcgcgcgcttcagcgccatccattttcggggctagttgattcggcaggtgagttgttacacactccttagcggatttcgacttccatgaccaccgtcctgctgtcttaatcgaccaacaccctttgtgggatctaggttagcgcgcagttgggcaccgtaacccggcttccggttcatcccgcatcgccagttctgcttaccaaaaatggcccacttggagctctcgattccctggcgcggctcaacggagcagccgcgccgtcctacctatttaaagtttgagaataggtcgagggcgttgcgcccccgatgcctctaatcattggctttacccgatagaactcgcacccgagctccagctatcctgagggaaacttcggagggaaccagctactagacggttcgattagtctttcgcccctatacccaagtcagacgaacgatttgcacgtcagtatcgctgcgggcctccaccagagtttcctctggcttcgccccgctcaggcatagttcaccatctttcgggtcccgacaggtatgctcactcgaacccttctcagaagatcaaggtcggtcggcggtgcacccctcgggggagatcgcgccggtcagcttccttgcgccttacgggtttactagcccgtcgactcgcacacatgtcagactccttggtccgtgtttcaagacgggtcgaatggggagcccgccggccagcaccgggagcacgcagttgccggagcacgccgagggcgcgcgctgtccgccacgatcggggcgacggcattccgcgggcatatcaattgcccgggctttggccgccgcctcaatccgcgctggtccacgccccgagtcgatcggcggaccggctctcgccgttccacatccgaccgggacgcaccgccggcccccatccgcttccctcccgacaatttcaagcactctttgactctcttttcaaagtccttttcatctttccctcgcggtacttgttcgctatcggtctctcgcccgtatttagccttggacggaatttaccgcccgattggggctgcattcccaaacaacccgactcgccgacagcgcctcgtggtgcgacagggtccgggcacgacggggctctcaccctctccggcaccccgttccaggggacttgggcccggtccgccgctgaggacgcttctccagactacaattcggacggcggggccgcccgattctcaagttgggctattcccggttcgctcgccgttactaggggaatcctcgtaagtttcttttcctccgcttattgatatgcttaaactcagcgggtaatcccgcctgacctggggtcgctgtcggaagcgctttcggcgccttggggtctttcgcgattcccgggcggacgacgcaccgaggcacgacggacagcaagcgggtcgagcaatcaaccaccactggtcgcgacgtgcgtcgccgggggatccgatttgggccggccgcgcggggacgcacgggaggccaacatccgccccccgccgcgcccgctccgttcgagcgggggaggggggggggcgacgcgatgcgtgacgcccaggcaggcgtgccctcggcccgaaggcttcgggcgcaacttgcgttcaaagactcgatggttcacgggattctgcaattcacaccaagtatcgcatttcgctacgttcttcatcgatgcgagagccgagatatccgttgccgagagtcgttttgacattgaggaggcgcgccgcaccccccgtccgcgcgcgccgcgtacggggcgggagaagggggccgggcgcttcgtttcggttttccttggcgctttccgcgccggggttcgttgccccacccgtcgcgccgggagggggggcagggccgacggcggggagcttccgccccgcgcgccgcggccccgccgtggttgcgcacgtgttcgcggtctgctctgcaggtttcgacaatgatccttccgcaggttcacctacggaaaccttgttacgacttctccttcctctaaatgataaggttcagtggacttctcgcgacgtcgcgggcagcgaaccgcccacgtcgccgcgatccgaacacttcaccggaccattcaatcggtaggagcgacgggcggtgtgtacaaagggcagggacgtagtcaatgcgagctgatgactcgcgtttactaggaattcctcgttgaagaccaacaattgcaatgatctatccccatcacgatgaaatttcaaagattacccgggcctgtcggccaaggctataaactcgttgaatacatcagtgtagcgcgcgtgcggcccagaacatctaagggcatcacagacctgttattgcctcaaacttccgcggcctaaaaggccgtagtccctctaagaagctggccgcggagggatacctccgcatagctagttagcaggctgaggtctcgttcgttaacggaattaaccagacaaatcgctccaccaactaagaacggccatgcaccaccacccatagaatcaagaaagagctctcagtctgtcaatccttgctatgtctggacctggtaagtttccccgtgttgagtcaaattaagccgcaggctccactcctggtggtgcccttccgtcaattcctttaagtttcagccttgcgaccatactccccccggaacccaaagactttgatttctcataaggtgccggcggagtcctaaaagtaacatccgccgatccctggtcggcatcgtttatggttgagactaggacggtatctgatcgtcttcgagcccccaactttcgttcttgattaatgaaaacatccttggcaaatgctttcgcagttgttcgtctttcataaatccaagaatttcacctctgactatgaaatacgaatgcccccgactgtccctgttaatcattactccgatcccgaaggccaacgtaataggaccgaaatcctataatgttatcccatgctaatgtatacagagcgtaggcttgctttgagcactctaatttcttcaaagtaacagcgccggaggcacgacccgaccagttaaggccaggagcgcatcgccggcagaagggacgagcggaccggtgcacacctggggcggaccggcccgcccaacccaaagtccaactacgagctttttaactgcaacaacttaaatatacgctattggagctggaattaccgcggctgctggcaccagacttgccctccaatggatcctcgttaagggatttagattgtactcattccaattaccagactcgtagagcccggtattgttatttattgtcactacctccccgtatcaggattgggtaatttgcgcgcctgctgccttccttggatgtggtagccgtttctcaggctccctctccggaatcgaaccctaattctccgtcacccgtcaccaccatagtaggccactatcctaccatcgaaagttgatagggcagaaatttgaatgatgcgtcgccggcacgagggccgtgcgatccgtcgagttatcatgaatcatcgcagcaacgggctgagcccgtgtcgaccttttatctaataaatgcatcccttccagaagtcggggtttgttgcacgtattagctctagaattactacggttatccgagtagcaggtaccatcaaacaaactataactgatttaatgagccattcgcagtttcacagtctgaattagttcatacttacacatgcatggcttaatctttgagacaagcatatgactactggcaggagatcggaagagcg

> *CYP* c249980_g1_i1

attccgagtgagaagaatcagtgagtccagaaatttctgtagcaaaatgcctaaccctaaggttttcttcgatatgaccgttggtggccagccggccggccggatcgtgatggagatctacgccgacgttgttccgaggacagcggagaacttccgcgcgctgtgcaccggcgagaaaggcgtcggcaaatctggcaagcctctctactacaaaggatcgaccttccaccgcgtgatcccgaatttcatgtgccagggaggcgatttcaccgccggaaatggaaccggaggcgagtcgatttacggagccaagttcgcggacgagaatttcgtgaagaagcacaccggtcccggagtgctctccatggcgaacgctggtcctggaaccaacggatcgcagttcttcatctgcaccgcgaagacggagtggctggacggcaagcacgtggtgttcggccaggttgtcgagggcttagacgtcgtgaagtcgatcgagaaagtgggatcgtcaagcggaaggacctcgaagccggtggcgatcgccgactgtggccagctctgatagatctatctcgtgttgatctgttgtgttatagatctgacgatgatctgtggtttgacggtcgccgctttttaccttttcgcttaattatctatctgcgttatccttctgctgtttttcagtcttatgagaggtggtattgtgtttgcccttcctttttttaatcggactatgcttctatttctcgactgtttttcttaataagatccatggatcgtttatcactgcttttgttaagctaattgttctttagatcttttgttttgttttgttctgttctggatactattattccgaacttaagttctcctgttgaaaatttcgactacaaaagaagtatcaacgtagacgttaacgaaattaacgttgaattttctgttgagttgtttcaacaacaataattacaaattgaacagcgtgctgtattcatggtttatgctcactctttttagtgaaatgacaccgaattattgtggtaattccagcgtgagattcgtgattgagtatgaactcggcaattattttcaagagatttgaattggtctaaactctcagaggatccgagtttatcggtggcatttgaataacaaaaaaaatgtattattatcatgagggcattgaattgcatttaaggaattcgaataactact

>*EFa*  c104359_g2_i2

cgacagaaaccctaattgcactcccttttcttcgttcgctatttatagagctatttggagtatgctccgtctctctccctctccgcacaagtcaaaaattttcttgcaagcacagccggcggcgagagagctccccttcactaattcctagccgaagctctctttttccaagtcaaatatgggtaaggaaaagactcacattaacattgtggttattggccatgtcgactctgggaagtcaaccactaccgggcacttgatctacaagcttggtggtattgacaagcgtgtgattgaaaggtttgagaaggaagctgctgagatgaacaaaaggtcattcaagtatgcttgggtgctggacaagctcaaggcagagcgtgagcgtggtatcaccattgatattgccttgtggaagtttgagaccaccaaatactactgcactgtcattgatgcccctggacatcgtgattttatcaagaacatgattactggaacatcacaggccgactgtgctgtcctcattattgactccacaactggtggttttgaagctggtatttctaaggatggtcagacccgtgagcatgcactgcttgctttcactcttggtgtcaaacaaatgatttgctgctgcaacaagatggatgccacaactccaaaatattccaaggcgaggtatgatgaaattgtgaaggaggtgtcttcctacctgaagaaggtcggctacaaccctgagaagatagcatttgtgcccatctctggttttgagggcgacaacatgattgagagatcaaccaaccttgactggtacaagggcccaactctcctggacgcacttgacatgattcaggagcccaagaggccatcagacaagcccctccgcctaccacttcaggatgtgtacaagattggtggtattggaactgtccctgtgggtcgtgttgagactggcgtcatcaagcctggtatgcttgtgacctttgctccaactggcttgaccactgaagttaagtctgttgagatgcaccatgaggccctgcaggaggcacttcctggcgacaatgtggggttcaatgttaagaatgttgctgttaaggatctgaagcgtggctttgtggcctcgaactccaaggatgatccggcaaaggaagctgctaacttcacctcccaggttatcatcatgaaccaccctggacagattggaaacggttatgccccagtgttggactgtcacacctcccacattgctgttaagtttgctgagcttaagaccaagattgacagacgatctggtaaggagctggagaaggagcccaagttcttgaagaatggtgatgccggtatggtgaagatgattcccaccaagcccatggtggttgagactttctctgagtacccaccactggggcgtttcgctgtgcgggacatgcgccaaactgttgcagtgggtgtgatcaagagtgtggagaagaaagacccatctggtgctaaggtgaccaaggctgctgccaagaagggagccaagtgaaccgtgcagatttttgattctggatcaagggaggtttatcttattaatgaaaaatactatcaagataccctattttctattattgcattatttggtcagacaatattttcatgctgcgttttgccttatttgttgagtggtcagtcccaaaactgggtgcttgacaggcggtggcatgagttaccggatgtgtctttttaagttttgtgttttttccattgggtggatcgtcaagtttgagtttatcttttattctgagtattaatattaatgcaattttagctgagttatatattactgcttaatcatacctgtggtttttatgttttgtcatgtatttt

>*MDH*  c127516_g1_i1

cttacagctaatttattttagcagtttctttttttcggccgttgaaaaacaaaaattagggcaaaacgtcacaggccacgggaacaacctatgcggcgtattgggttgtaggtgaaatgtcaaatgtaaccctgatcaggtcagaaagcattcataaatatcatcagtatcagtactgttgtcgaaacccaagtcactcactccctctgaactctctcgaaaccctcttcgttttcatcgcttttcagatcggagatccacttcgtggaaatggccaaagatccagttcgtgttctcgtcaccggtgccgcgggtcaaattggatatgctcttgtcccgatggttgccagaggagttatgttgggtccagatcaacctgtgatcctccacatgttggatatagcacctgctgctgaggctcttcagggtgttaaaatggaattgatagatgcagcatatcctcttcttaaaggtgttgttgctacaacggatgctgttgaagcttgcactggtgtaaacattgctgtcatggttggtggattcccaaggaaagagggcatggagaggaaagacgtgatgtccaagaatgtctcaatttacaagtctcaagcttctgctcttgagaagcatgccgctgcaaactgcaaagttttggttgttgcaaaccccgccaacaccaatgcattgattttgaaggaatttgctccatccatcccagagaagaacatcacatgtttgactagattggaccataacagggcccttgggcaggtctccgagagattgaatgttcatgtgtcggatgttaagaacgtcatcatttggggaaatcattcctcatcacaataccctgatgtcaaccacgccaccgtgaaaactccatctggggagaagtctgtccgtgagcttgttgctgatgatgcatggttgaattcagagttcataaccattgtccagcaacgtggagctgctataatcaaggcgaggaagctttccagtgcgctttcggctgctagctctgcttgtgaccacatccgtgactgggttcttggaactccagagggcacatgggtttctatgggggtgtactctgatggttcatacagtgtcccctctggactcatctattctttccccgtcacttgcaaaaacggtgaatggaccattgttcaaggccttccgatcgatgagttttcaaggaagaagctggatttgactgcacaagagctgagcgaggagaaggccctggcatactcctgcctgtcttagattccatactggatttgactacaccagagatgagtgaggtgaagatcctgtcttaaattccatggtggtgccatatcgtaagcagtgtttccatttgtttcgcgactgcagttttgaataatccatagttaatttgttttcccactattaatggtggaaaactactttctgtgatcatccatcctcaaggacttttatggtgtatgagatgagtttttcaatttgacaggacttttatcatatgagattctacatctaattatcatttgtactggttttctacttaggtgggattgcgaaaaatatactctacctcatcattttaaaataaggaacattcttttgtacattaacaatgtccattcttttaaaaaaaatggagggagcgctacatatctggcctatggattagtttggcctgactgaatggaattaatgaaaattacagtattgtagtataaaaatgttagagaaagagaaataagtttgtgga

>*TUBa* c70526_g1_i

aaaagaaaaagaaaaagaaaaagggaagaaacccagacacagaaaacccattaattgaaatgatgatatgatggggcattattcaccaggagcctcctccagccaacattccatttaaccagtatccactggtccccccttgccctctttcttgtccaaattcattgcttaaaggaaccctcttcacttcctcaatggatgatgacaaattcatctcatgatcattcacatacccgcctccattaatctcctccactggaaaccccaaattcatcttcacatcaccgatcataggactattaaacccacaaggaacggacactacaccgccctgatccgacacaggcgccataactgggaaataatcatgaagagggttccgattgcaaaggttagggttttggatcccaaatgggtagggattgatcaaactcaaattcagctgatgatcaatcatcatcctacagttttggttatgagaagcagcaaataatgggggtgggctgccgccgccgctgctagttagagatgatttcttcgacaagggctgttcaggacagtaattatttggattgttaaaaataggtcttttgtggaggctctttctagaacctccaccaacagggacgtttcttagggtgcccccttctgtccaataccttctacaagccttgcaaaagtaccttggctgggaaaggctgtagttgttgtaataacagaatttggtgttgctggaattgcaccttcgacaattcaacgcccgttttgtttccttttcacccctgctaatgtttcccttcttttctccatttccagacgttcctgatgccttctcaattacattaaccccaattccctgtggccactgagtagaagtatgactatccataagcgcacaaccaaaaggaggagagagacagagagaaagagaaagagaaagagaaagtgagatagagatagagtgtagagagagtacattgtaatactgtctctatagtctctacccatttctttgatttgatttagtagtgctacttcaccttctaaaaactagaattaacagcagatcgggtcagatctactcatctacacactacacccaacacaagatgagagaaatcatcagcattcacattggtcaggccgggatccaagtcggcaactcttgctgggaactctactgcctcgaacatggcattcaccctgacggcatcatgcccagcgacacaaccgtcggcgtcgcccacgacgctttcaacaccttcttcagcgagaccggctccggcaagcacgtcccccgcgccatcttcgtcgatctcgagcccaccgtcatcgacgagatccgcaccgggagctaccgccagctcttccacccggagcagctcatctccggcaaggaggacgccgccaacaatttcgcccgtggccactacaccgtcgggaaagagattgtcgatctctgccttgatagggttaggaagctcgccgataactgcactggactccaaggcttcctggttttcaatgctgtcggtggcggcactggctccgggttgggatccctgctcctggagcgcctgtcggtggactacggcaagaagtccaaattagggtttacgatctacccttctccccaggtctccacggcagtggtcgagccctacaacagtgtgctctccacccactcccttctcgagcacaccgatgtggcggtcctcctcgataatgaggccatttacgacatctgccgtaggtcgttggacatcgagagacctacatacaccaatctcaaccggctgatttcgcagattatatcctccctcaccacttccctccgctttgatggcgccatcaatgttgatatcactgagttccagaccaatctcgtcccttaccctcgtatccatttcatgctttcatcctacgcgcccgtgatctctgccgagaaggcgtaccacgaacagctgtcggtgcccgagatcaccaatgccgtgtttgagccgtcaagcatgatggccaaatgtgatcccaggcatgggaagtacatggcttgttgtttgatgtaccgaggcgatgttgtgcccaaggatgtcaatgctgccgtcgccaccatcaagacaaagaggactgtgcagtttgtggattggtgccctaccggattcaagtgtggaatcaactatcagccaccggcagtggttcctggaggggatcttgccaaagtgcagcgtgcggtgtgcatgatcagcaacaacacagcagttgctgaagtgttctcccgcattgaccacaaatttgatctcatgtatgccaagagggcatttgtccactggtatgtcggtgaaggaatggaggaaggggaattcagcgaggcccgggaggatctggctgctctggagaaggactatgaagaagttgggatggagggtgtcgatgaggaggaggaaggcgacgagtactgatgatgagggcggtccggcattgcgttgtgcccatcttgtgtatcatttatttggatttgttgtgtcattaatattcccctctatggtatgtttatggttcttatttggaatgttttgctgattattgttctgagacttcctttacatttgtgctatatgtatgttgtgacttgtgagagttatatgccatcttattcttatgtagaatctatcggtggtgttgtctgtcgttgagtcgagatgagatccctttgtatactatatccatccaacagtttatcagagtatctgtttgtgaaatgtgaaatgtgaaatgtgatgcagattcctcgaacatatatactgatggtagtaattactagttgatacttgaaaggtattggatcaaatatca

>*TUBb* c52210_g1_i1

ccaatcagacaattctatttccaccttcatatcctcttccttcattacaaaacccaattaaactttcatccgtattcattctcgatctcttccaacttccaagttatagtaccgtaatctattttcagagtgtaatagaagatcaaagatgagagaaattcttcatattcaagcagggcagtgtggaaatcagattggtggcaagttttgggaggtggtttgtgaagaacacggtatcgatgcccttgggacctacactggcaattccagggttcagcttgagagggtgaatgtttactacaatgaggcgagtggggggaggtatgtcccgagggctgtgttgatggatctggagccagggacgatggacagcctaaggacaggaccatatgggaagatctttcgaccggataactttgttttcgggcagaacggtgctggaaacaattgggcaaaggggcattacactgaaggagctgagttgattgactctgtgctagatgttgtccgaaaggaggcggagaattgtgactgcctacaagggtttcaagtgtgccattcacttggaggaggcactggatccggaatgggaacactcctgatctcaaagatcagagaagagtatcccgacaggatgatgatgacgttttcggtcttcccctcacctaaggtctctgacaccgttgttgaaccctacaatgccaccctctcagttcaccaactagtggaaaatgcagatgaatgtatggtcctagacaatgaagccctctacgacatctgtttccgcacgctcaagctcactaatccaagcttcggtgatctgaatcatctgatatcaacaacaatgagcggcgtaacctgttgcctccggttccccggccagctgaattccgacctccgcaaactagccgttaatctcatcccattcccgcgcctccacttcttcatggtagggttcgcgccgctaacctcgcgcggctcccagcaatatcgtgccctcacaattccggagctcacgcagcagatgtgggacgcgaaaaacatgatgtgcgccgccgacccccgccacgggcgctatctgacggcctccgccatgttccgcggaaagatgagcaccaaggaggtggacgagcagatgatcaacgtccagaacaagaactcatcctattttgttgaatggatccccaacaatgtgaagtccacagtgtgtgacattccaccaactggtttgaaaatggcgtccacatttattgggaactcaacttcgatccaggagatgttccggagggtgagcgagcagttcacagccatgttcaggagaaaggctttcttgcattggtacacgggagagggaatggatgagatggagtttactgaggcggagagcaacatgaatgacttggtgtctgagtatcaacagtatcaagatgctacagccgatgaggacgaatactatcaagaagatgaggaaattcaggacgaagtttaatcatttttattgtatttttactcgttgatgtctgtgtgctgagtaattatgctttctactgggtattgtgttttacctgcattcttggcatgtttgctatatggtttttggggtgtcttttttgaagttgttagtgtgtttgaatgttaatggagtgagtgtattagtaggggggtttgtgaattgttttttttggtgattataaactttcctttcagattgatatttcatgccaactaaatcttcttttgccaaactgtgctatgaacaatacaaatttgataaaactaaagaatccattaattaatgttcacgtctcagaaaatctacaaggatgatgcacatcaataatcaccacacaccctagcaagtagcaacccaaacccagacgcaaaaataaaaaccaagttattaagcataggcagatataaaaagcaggatggagttcgtctgctcctccttttctcaacaacagtttaagcaccaaacttagggaccttagcagtagatatctgcgcaaggaagtgttccgacacaatccgacgctggattttcccagttgcagtttttggaagcgagtctgtgatgaaaacctgctttggaaccttgaaagcagcaagatttttcttgcagaacctggagacctctgcctcatccaggttcgatccttctctggggattacagcacagtttatttcttccccatatttatcatctgggacaccaaatgctacagcctgagcaatatcgggatgggacaacaggacggcatctacctcaattggtgagatcttctcccccccacggttgatcaattccttgattcggccaacaagatgcaaatatccatctgaatcaaaataccccaaatctccagtgtggaaccaaccaaactggaaagcagttttattggcctccgggttgttcttgtacccttttgtgacattagagccccttatgcaaacctctccatttgaattaggtccttgaagcacaccactttcatccaatatacccatctcctgaccgataggtttacccactgatccaggcttatgtggaccatcctccggcaaagggttcgacgccatcaaatgtgttgcttctgtcattgcataagcctccaagaccggtgcttcaaatgcctcctccagccgagccaggattgcaggggccaaagatgcgctacagcttcggatgaagcgaagctttgggtaggatgattcagggcggctaaggtgtcggtcgagtatgatttgatgaatggttggcacggcagtgtaccaagtggcgctatactttttcatatcagaccagaatgttgaagctgagaatctaccggcagctggcagagccacagcagcaccggccccaagtgagcttaataagccagccaacaagccgtgcacgtgaaacaatggaagaacaatcaccgtggagtccgcttccgtcagtttgtagacagatttgatattttggacagaggaatacagattttcctgagttaggggtacgcctttgggtcggctggtggtgccggatgtgtggaggaaaagtgcaacgtcggaggggtcattggtgagtttcgagagcgaatcagtatcaagctcggactgagttgctggagataatataatttctgaatcagcggatggcaacgcggcggagaggtgaggtatgttgagctttgtggctgctgcttgagccggctcgttgccttcttttgatgtgagtaacagctttgattctgagtctgataagtagaactcgaactcatcaaaagtgtaggcagagttgagcggcgccgccgttgctcgggctcgtagcacgcccaaaaacattatgacaaactcaatagtgtttgggaacgtgagagcaacaacatccccaggcttcactccagcagccacgaggcgagcggcggcgtgttctacgtgttgaagcagttccgcatgtgtgagatcgaagcttccggatacggagatagcgcggcgggaggggaatagtccggcgacgtggctcaacaatccggtgagagtgggcgtagaagccattggaaatggaaaccgttgggaaaattctagagttaaaaaaaaaatgtttaacaaaatgaataaagaaatgaaagatatgtatgaattgaatggaggtgataatatgggacgcgatatatatatatagagacaggggacggagacacggagaaactcagaaacgggatttcgactttcgac

>*UBC* c187724_g1_i1

gagggcaaagcacccacattccctagttgcctcttgtgcttcttaggtcttcgtcaatcggccacctttttattcctcttggtctcgtcaatcgcttctccctctctcgccgaaggttttttagctttccgacagagaaacactttgatggcttcaaagagaatccagaaagaattgaaggatttgcagaaggaccctccggcctcgtgcagtgctgggccggttggacaggatatgttccattggcaggctaccattatgggtcccaccgacagcccatttgctggaggagtgtttctagtaatgattcacttcccacctgattatccctttaaaccacccaaggtctctttcaaaactaaggtttatcatccaaatatcaacaacaatggcagtatttgcctcgatatcctgaaagaccagtggagccctgccctcactgtttccaaggtgttgctctcgatctgctccttgttgactgatccaaacccagatgaccctctcgtgcccgagattgcacacctatacaagactgacaaagccaagtatgaggccactgctcgatcatggacccagaagtatgccatgggataaaatgcttgctgttgggactcagcggcactgtgtttgactagtgataaatacaataaatatagataaagaaggatatgatattttacttttgctttattattacttatgtttgcgattcctggtgttggatcgaaataaaatttattagtggtattattattgttttttaggacatttgtgggatggataaaatttatgattttaatatttttgggattgcgatggtttgtatgggttacttggctttgtttgattcttcaacaaaccattgttctatgcctaaatattgtatttccttctttttcctttttatttctgttgctacgctttttttattttataaatttacaaatgagcgtagcaaataaaagttgaaaaaacaaagtaaggatgttgctaagaaatatattcctcattaattagcctatcatattattagatggacgatggagatcggaagagc

>*UBQ* c105454_g1_i1

aaaaattgctttggatgacaactgggggaataaatgtagggcccatgttataaaggcccaatatctgaaaatggcccaatagccggtccataaatagcactacgcattcagctgccacgaacaatctcaattcacaaagaggttctccaatttcagctttgaggagcagtgttggagtttgagaatctcagagagccacgtacaaatctagcagggtaggggaaaacatgcaaattttcgtcaaaaaactgggcggagaaaccaccgctcttgatgttgagtgcactcataccaccgaagctgtcaaggccatgattcaggacaaagaggagtcatttatcggccagcagaggcttgtgtttgctggtaaagtattggatgatgggagacctttgtctgattataacattggaaaagagtcaacaatttaccttcatttgaggcttagaggaggaacgatgatcaaggttaaaacccttaccggaaaagaaatcgagattgatatcgagccaactgatagcattgaccggattaaggaacgggtagaggagaaagaagggattcctccggtgcagcaaaggctcatttacgcgggcaagcagcttgcagacgacaaaactgccaaagactacaacatagagggtggctctgttcttcacttggttcttgctctcaggggtggcagtgtgtaagtagctctatatatatgcccggggaattcatggcctactactatgattttaaatgtactatcgaaaaaactgcgaattgtgatgaaacccaggtttgattttagtcgggaatcgaaatttatgtttcctttatgtgctagactgtcattcgtcgtgcatctttagttgtttctatgataatttggattcctctgctgttttggtcatgatcaatgagttaagatggctttcgcggattgctgttggtgtcccaactcccatagtatggtcagctgaggtaccattgttatgacattttgaaagttttcttgtgctattttaatttttatggggtgttgggttgttgagaaagacttatttgtgaactctgctggacagtggacactactctttgttaaaaacctttttaaacttacgtgtgttgatttggtctattttgctgcacttgcatactatggttagtacttagtactaccatatttatatttttaataggggatgaaagcaaatttatacacttaatatctatttatatctctcaacttttttcttcgcttaattatttattttcatatacaatttttttgtttctctcagagcatccaccaatccccg

>*ACT* c103390_g1_i1

gccgttcagctatatcaaccattaatgtgatgcaataatgggtgccacaaataagagatctggagcattcaatccgacggctcagattataggaacatgaatttgagctgaatttgagctgaatttcatttgtgtctgagtttcatttcccgtgttttgttttaaacgcgagggccgcgacctaaattcaaggaacaacgcacagtccaacgttatcgaggggacaagctgagaggatttctccgcaccacccaaacccattgccaccaacccctcaaaaggtaatatttaagagatggccgaaactgaggatattcagccccttgtttgcgacaatggaactggaatggtcaaggctggatttgctggagatgatgctcctagggctgttttccccagcatagtgggacgtcctcgtcacactggagtcatggttggtatgggccaaaaagatgcatatgttggtgatgaggctcaatccaaaagaggtattctaacattgaagtatccgattgagcatggaattgtcagcaactgggatgatatggagaagatttggcatcataccttctacaacgagcttcgtgtggccccagaagagcacccaatcctcttgacagaagctcctctgaacccaaaggccaatcgtgaaaagatgactcagatcatgtttgagacctttaacgcccctgctatgtatgttgctattcaggctgttctttccctttatgccagtggtcgtacaaccggtattgttctcgattctggtgatggtgttagccatactgtccccatctacgagggttatgctcttccccatgcgattcttcgtcttgatcttgctggtcgtgacctcaccgattacctcatgaagattctgactgagagaggctactctttcaccactacagcagagcgggaaattgtgagggacatcaaagagaagctatcttacattgctcttgactatgagcaagaactagaaacagccaagacaagctctgctgtggagaagaactacgaactgcctgatggacaggtgattaccattggtgctgagaggttcagatgccctgaagtcctgttccagccatccatgatcggaatggaagctgctggcattcatgaaaccacctacaattctatcatgaagtgtgatgtggatatcaggaaggatctctatggaaacatcgtgctcagtggtggaacaactatgttcccaggcattgctgatcgtatgagtaaagaaataacagcacttgctccaagcagcatgaagattaaggtggtcgcaccacccgagagaaagtacagtgtctggattggaggatcaatcctggcatccctcagtactttccagcagatgtggatctcaaaggcagagtatgacgaatccggtcctgcgattgttcacagaaagtgtttctaagttgggacaacatttcttattatccacagaagtttgaatacagtaagtgcgtgtgtgggttttggattgcagttttgaatatgaagtagtggtgatgttcatgagtggggttgttttgttattacttattagaaatgcgtatgctgatttgtgctctatggcatattggtaggaaattatatgtctgtacctcagcagctgtaatatttgggtagtttttactttttggttttgtttgtttttttatttttatttttatttttttagaatatggtttaattgctatttgctactagtgttaatgccatatttgaaaattgagtgggatttggactttggatttgtggaagttgctttcttcacaacattactatattgaaaaatgagatacagtgggg

>*GAPDH* c218573_g1_i1

ctcagatcagcaaggctttaactgacacagtcacagataaaaaggatttgatgaaaaagctacccaagtttatctacgatgaggaaaaggcccttgagagacagcggaagaaactggctgagaagattgaacaactcaactctgctatagacaacgtctccaatcagctgcgaccagaagaaactccagatgaggcagcagtcagtccagacgaatttgaagttgctgtgtaaatggctgctacatttggcccacttatgcaatctcattgtttggttctgatgacaaatgtcatctgggatgctgtttttttgaccttgggtcattgtttcaaactttcagagctgagtattatgagatgttagttcaatgtctgttcgtgttgatgatttccatgttttgaatattagtactttgtgccgcctctctacaggagtatactcttcaatctgataaaaacacttttgtctgtagatttccactatactacggttgtctgtactgtgagattttgtactcttccctgcaataatgtgattttcattgtcttgaaggatacacacgtcattttaactatggcttgaggaagatattactcctatgatgattatccatctcccagaagaggaaaatagtgacaaatatattctattggttcaggctttttctttgacactggtggtaaatgatatgttgagggtactgttcaaatgtgggtgaaaagggcttggagggaaaatggacaaatgaaggaacagtgattggtgaaaccattctgtggttcatatttttgggtggcacagaaattagacatgatcttatctgatatcaatcttcacactccataatcaccactcactgctgctgactcaattccaaccataactttgttccaattcaagccagaaaagaaaaaaagtaataacgatcgctaagagtataaagcattgcacacctcaaatatagctttgaatttttgtatgtaggtatttttgcagtttccttgcagccatggcatcgtcccacgcagctttggcatcttccagaatcccgacaaccaccaggtttccttccaaggcggctcattcttttccggctcaatgcttctctaagaagcttgagatagcagaattctcagggcttaggtctagtggatgtgtgacctacgccaagaatgccaaggaggcttctttctttgatgcggtagcttcccagctcgctccaaagactgcaggatcagcacccgttaagggaccaactgttgcgaaactgagagtagctataaatggtttcggacgcattggtaggaacttcctccgctgctggcatggccgcaaggactcacctttggaggtcattgtggtgaacgacagtggtggtgtaaaaaatgcctcgcacttgctcaagtatgactcaatgctcggcaccttcaaggcagatgtcaaggtagtcgacaacgagaccatcagcgtagatgggaagcccataaaggttgtctccaacagggatcccctcaaacttccttgggctgaacttggaattgacattgtcattgaggggacaggtgtttttgtggatggtccaggagctggcaagcacatccaagctggagccaagaaggttatcattactgctccagcgaaaggggctgatattccaacttatgttgttggcgtaaacgagcaggactactctcacgtggttgctaacatcataagcaatgcttcttgcaccaccaactgcttggctcctttcgttaaggtcatggacgaagaatttgggattgtcaagggcacaatgaccaccacacactcatacactggggatcaaaggcttttggacgcgtcacaccgggacctgagaagagccagagcagcagcattgaacattgtgccaacaagcaccggtgcagccaaggccgtatctcttgtgctacctcagctcaagggcaagttgaatggcattgctctccgtgtgccaacacctaatgtatcagtcgttgacctcgttgtgaatgttgagaagaaaggaatctcagctgaagatgtcaacaatgccttcagaaaggcagctgatgggccattggatggagtactggctgtgtgcgatgtccctctcgtctcagttgacttccgatgcagcgacgtttcctccacaatagattcatcactgacaatggtgatgggagatgacatggtcaaggtggtggcctggtacgacaatgaatggggatacagccaaagggtcgtggatttggcacatttggtggctgctaagtggcccgggcaacctgcaaaagggagcggagacgcgttggaggactactgcgagaccagccctgcggacaaggagtgcaaagtttatgaagcttaataagcttaagttcattcttgaattaaatttgggtagtaaattattatcagtgcatctgtgaaacatttgttgaagttcaatacatatatatatatgagagagacacattggaatattggatattaaggtcaagttccactctttccttatatacccttttacatgttcaatcgcttgcttcatcacatacattcacaaggtttaaaaatttaaataaaaaaatcatactgcc
